# RNA Metabolism and the Role of Small RNAs in Regulating Multiple Aspects of RNA Metabolism

**DOI:** 10.3390/ncrna11010001

**Published:** 2024-12-24

**Authors:** Pranav Dawar, Indra Adhikari, Swarupa Nanda Mandal, Bhumika Jayee

**Affiliations:** 1 Department of Biological Sciences, Texas Tech University, Lubbock, TX 79409, USA; iadhikar@ttu.edu; 2 Department of Plant Sciences, University of California, Davis, CA 95616, USA; snmandal@ucdavis.edu; 3 Department of Chemistry and Chemical Biology, Northeastern University, Boston, MA 02115, USA; b.jayee@northeastern.edu

**Keywords:** mRNA, small RNAs, non-coding RNAs, Transcription, TGS, PTGS, mRNA decay, RNA processing, RNA modification, RNA editing, Retrograde signaling

## Abstract

RNA metabolism is focused on RNA molecules and encompasses all the crucial processes an RNA molecule may or will undergo throughout its life cycle. It is an essential cellular process that allows all cells to function effectively. The transcriptomic landscape of a cell is shaped by the processes such as RNA biosynthesis, maturation (RNA processing, folding, and modification), intra- and inter-cellular transport, transcriptional and post-transcriptional regulation, modification, catabolic decay, and retrograde signaling, all of which are interconnected and are essential for cellular RNA homeostasis. In eukaryotes, sRNAs, typically 20–31 nucleotides in length, are a class of ncRNAs found to function as nodes in various gene regulatory networks. sRNAs are known to play significant roles in regulating RNA population at the transcriptional, post-transcriptional, and translational levels. Along with sRNAs, such as miRNAs, siRNAs, and piRNAs, new categories of ncRNAs, i.e., lncRNAs and circRNAs, also contribute to RNA metabolism regulation in eukaryotes. In plants, various genetic screens have demonstrated that sRNA biogenesis mutants, as well as RNA metabolism pathway mutants, exhibit similar growth and development defects, misregulated primary and secondary metabolism, as well as impaired stress response. In addition, sRNAs are both the “products” and the “regulators” in broad RNA metabolism networks; gene regulatory networks involving sRNAs form autoregulatory loops that affect the expression of both sRNA and the respective target. This review examines the interconnected aspects of RNA metabolism with sRNA regulatory pathways in plants. It also explores the potential conservation of these pathways across different kingdoms, particularly in plants and animals. Additionally, the review highlights how cellular RNA homeostasis directly impacts adaptive responses to environmental changes as well as different developmental aspects in plants.

## 1. Introduction

RNA metabolism is an essential cellular process that allows cells to function effectively. RNA metabolism, as the name implies, focuses on Ribonucleic Acid (RNA) molecules and includes all the critical processes an RNA molecule may or will go through throughout its life cycle [1,2,3,4,5,6,7]. Various processes like RNA polymerase-mediated RNA synthesis, maturation (RNA processing, folding, and modification), intra- and inter-cellular transport, translation, and catabolic decay together shape up the transcriptomic landscape of a cell at a given time and state [8]. Other than RNA molecules present in the nucleus, RNA metabolic processes also extend to the RNA population present in chloroplasts and mitochondria. In addition to RNA-binding proteins, biomolecules like metabolites, as well as RNA itself, determine the fate of RNA populations in a spatio-temporal manner.

**Figure 1:**
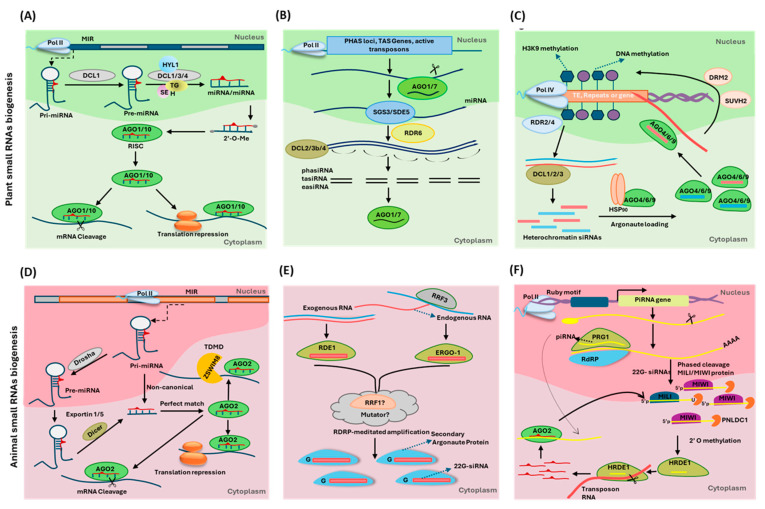
sRNA biogenesis models for plants and animals. (**A**) miRNA biogenesis pathway—RNAPII transcribes the *MIR* specific. Primary miRNA (pri-miRNA) is then spliced into a single hairpin structure by DICER-LIKE 1, which furthermore gets cleaved into a miRNA duplex of 21 nt with the help of nuclear dicing bodies (D-bodies) including DICER-LIKE 1/3/4 (DCL1/3/4), HYPONASTIC LEAVES 1 (HYL1), SERRATE (SE), and TOUGH (TGH). The duplex is then methylated at the 3′ ends catalyzed with HUA ENHANCER1. With the assistance of HEAT SHOCK PROTEIN70/90 (HSP70/90), the mature miRNA gets loaded into the ARGONAUTE1/10 (AGO1/10) (also known as RISC) based on the specific miRNAs. (**B**) Secondary siRNAs (phasiRNA, tasiRNA, and easiRNA) biogenesis model—After PHAS loci, TAS loci, and active transposons are transcribed via RNAP II, AGO1/7-loaded mature miRNA cleaves the transcribed mRNA. 5′ fragment of the cleaved mRNA degrades, while the 3′ strand is converted into a double strand with the help of RDR6. SGS3 and SDE5 help in recruiting RDR6 to the recognition site. DCL4/3 participates in ta-siRNA and phasiRNA production, whereas DCL2/4 participates in easiRNA production. The 21–24 nt mature siRNA strand then gets loaded into AGO1/7 for downstream gene regulation. (**C**) Plant endogenous siRNA biogenesis—The transposable elements, repetitive regions, or gene introns get transcribed with RNAP IV, which then gets converted into dsRNA with the help of RDR2/4. The dsRNA then gets cleaved into 20–24 nt fragments with the help of DCL2/3/4. HSP90 then helps to load the mature siRNA strand in the respective AGO4/6/9, based on their origin. (**D**) Canonical and miRNA Biogenesis in Animals—After the transcription of the *MIR*-specific locus with DNA-dependent RNA polymerase II, pri-miRNAs were converted to single hairpin-like structures (pre-miRNAs) with the help of Drosha. The pre-miRNA transported into the cytoplasm with Exportin1/5 proteins then gets further cleaved into miRNA duplexes by Dicer proteins. The pathways create this miRNA duplex without Dorsal/Dicer acting upon the primary miRNA. The mature miRNA strand then gets loaded in the AGO2. (**E**) Endo- and exogenous modes of siRNA production in *Caenorhabditis elegans*—siRNAs derived from ssRNA, and dsRNA are loaded into primary Argonaute proteins, ERGO-1 and RDE1, respectively. The loaded primary Argonaute protein, with the help of RRF1 and Mutator, mediates the conversion of 26 nt long 5′-guanosine siRNA into 22G siRNA. The produced siRNAs are then loaded into the secondary Argonaute proteins for downstream gene silencing. (**F**) Biogenesis of piRNA and the regulatory ping pong cycle of biogenesis in *Drosophila melanogaster*—*piRNA* gene sequences are marked by an upstream Ruby motif. The piRNA precursors are transcribed by RNAP II and then exported to the cytoplasm. These precursors are then processed by endonuclease Zucchini and an unknown 3′–5′ exonuclease. Via DmHen1/Pimet methyltransferase, the 3′ end of the mature piRNA gets 2′-O-methylated. The mature piRNA gets loaded into PIWI, forming piRISC to regulate methylation of TEs. Apart from PIWI protein alone, some gets loaded into Aub, which then initiates the ping pong cycle of biogenesis. Aub loaded with piRNA and AGO3 loaded with secondary piRNA repress TE activity through DNA cytosine methylation. PIWI-related Gene 1 (PRG-1) is required for primary piRNA activity, whereas HRDE-1 (Heritable RNA interference (RNAi) deficient protein-1) is the Argonaute protein that carries RdRP-amplified 22 nt, 5′-guanosine siRNA (22-GsiRNA). (Modified from [16]).

**Figure 2:**
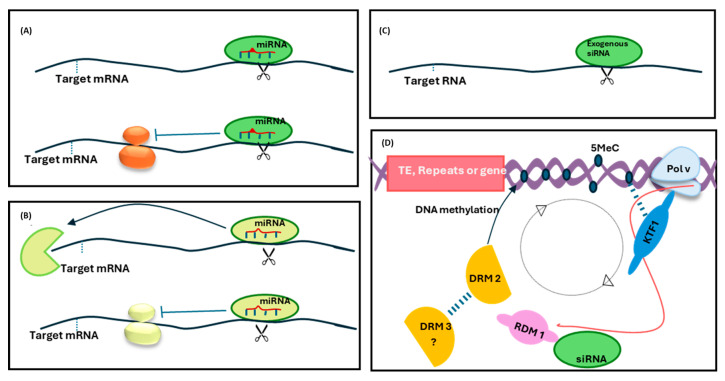
PTGS and TGS Mode of Action for miRNA, siRNA, and piRNA in Plants and Animals. (**A**,**B**) Post-transcriptional gene silencing of plant and animal miRNAs through mRNA cleavage, RNA decay, and translational repression. (**C**) Exogenous siRNA is only able to participate in PTGS through mRNA cleavage. (**D**) Plant and animals endogenous siRNA and piRNAs, the loaded AGO protein, after being transported back to the nucleus, target nascent RNA Pol-V transcripts (line represented in red) through complementary siRNA and form the RdDM complex (RNA-dependent DNA methylation). GW/WG protein, associated with RNAP V, KTF1 acts as an organizer by coordinating with AGO and 5-meC (5-methylCytosine). Similarly, the AGO-associated protein RDM1 interacts with DRM2, a RdDM complex catalytically active de novo methyltransferase, and binds with single-stranded methylated DNA. DRM3, a catalytically inactive paralogue of DRM2, is also known to be involved in the RdDM complex, but its function is still unknown. After all these proteins are localized, DRM2 catalyzes methylation of cytosine in all sequence contexts. (Modified from [16,18]).

Expanding on the complexity of RNA-mediated regulation of RNA metabolism, non-coding RNAs (ncRNAs), especially small RNAs (sRNAs), long non-coding RNAs (lncRNAs), and circular RNAs (circRNAs), play a major role in regulating the expression of RNA molecules in the living cells, often including their respective precursors, thereby forming a feedback loop [9]. In eukaryotes, sRNA-mediated gene silencing is a conserved mechanism that regulates gene expression at transcriptional and post-transcriptional levels [10,11,12,13]. Based on their origin, sRNAs are primarily classified as small interfering RNAs (siRNAs), derived from double-strand RNA (dsRNA) with perfect base complementarity, and microRNAs (miRNAs), derived from hairpin RNA (hpRNA) with some mismatches/bulges [14]. The miRNA, siRNA, and exogenic RNA silencing pathways are the three distinct, broadly defined RNA silencing pathways in plants and animals. In plants, the siRNA pathway can be further classified into three pathways: 21/22 nt miRNA-mediated tasiRNA (TRANS ACTING SMALL-INTERFERING LOCUS/TAS-derived siRNA), 21/22 nt siRNA-mediated phasiRNA (Phased siRNA), which leads to the formation of 21/24 nt long siRNAs, and 24 nt siRNA-mediated RNA-directed DNA methylation (RdDM) [14,15,16]. In plants, the miRNA, tasiRNA, phasiRNA, and RdDM pathways work together to maintain the RNA flux through the Transcriptional Gene Silencing (TGS) and Post-Transcription Gene Silencing (PTGS) activities (Figure 1) [15,16,17]. In addition to the miRNA pathway and the RNA-directed RNA polymerase family member 3 (RRF-3) mediated 26G siRNA endogenous pathways, another siRNA pathway known as piRNA (PIWI-interacting RNA) has been identified in animal systems (Figure 1) [16]. The piRNA pathway generates siRNAs ranging in length from 24–31 nt that interact with the PIWI-related gene 1 protein (PRG-1) and heritable RNA interference (RNAi) deficient protein 1 (HRDE-1), which target and silence transposable elements (TEs) via TGS or PTGS pathways (Figure 1 and Figure 2) [16,18].

Other non-coding RNAs, like long non-coding RNAs (lncRNAs) and circular RNAs (circRNAs), have been identified as an additional layer in regulating miRNA-target interactions. LncRNAs, such as *INDUCED BY PHOSPHATE STARVATION1* (*IPS1*), act as target mimics or sponges, providing a non-cleavable target for miRNAs (for example, miR399) through the presence of miRNA complementary sites. This results in an overall reduction of mature miRNA concentration in the cellular space, thus fine-tuning the expression of miRNA target(s) (for example, *PHOSPHATE2* (*PHO2*)) [19,20]. LncRNAs such as *GUARDIN* and *PNUTS* are also known to possess multiple binding sites of miR-23a and miR-205, respectively, in animal systems [21,22]. Similarly, circRNA CDR1 antisense RNA (CDR1as), expressed in human and mouse brains, acts as a sponge for miR-7 in the respective tissues [23]. Apart from regulating endogenous gene expression, sRNA-mediated RNA silencing also serves as a defense mechanism in presumably evolved by eukaryotic organisms against invasive nucleic acids (like transposons and transgenes), viruses, pathogens, and parasites [24,25,26,27,28]. The RNA silencing machinery has been utilized in plant and animal biotechnology for various engineered objectives, such as improving disease resistance, enhancing commercial traits, modifying flowering time, and developing therapeutics for cancer, neurodegeneration, and cardiovascular diseases [2,25,29,30].

RNA metabolism plays a crucial role in regulating gene expression at the transcriptional, post-transcriptional, and translational levels across all stages of development as well as in response to biotic and abiotic stimuli [1,5,6,31,32]. Among the common RNA metabolic pathways, transcription, nascent RNA modifications and processing, mRNA decay, and RNA editing are some of the most explored and established pathways in plants and animals. This review provides an overview of the current understanding of the various facets of RNA metabolism and how sRNAs, primarily miRNAs and siRNAs, influence these intricate and multi-step regulatory networks responsible for the regulation of gene expression.

## 2. Eukaryotic Transcriptional Machinery and sRNA-Mediated Regulation of Transcriptional Dynamics

The biological mechanism underlying the ‘molecular dogma’, through which genetic information is transferred from DNA to RNA, is known as transcription. The regulation of transcription in plants and animals involves the integration of various internal and environmental cues [33,34]. Genome-wide chromatin immunoprecipitation techniques have yielded valuable knowledge regarding the transcriptional machinery, interacting components, and network topology involved in developmental phase changes and cellular differentiation [35,36]. Transcription is a complex biological process that can be categorized into three distinct stages, namely **initiation**, **elongation**, and **termination**. The process of transcribing protein-coding genes in eukaryotes is executed by 12-subunit RNA polymerase II (RNAPII), which is a crucial and meticulously controlled aspect of eukaryotic gene expression. Transcription in eukaryotes is primarily regulated by diverse sets of transcription factors (TFs) (general and specific TFs; general TFs (GTFs) like TFIIB, D, F, E, and H), TATA binding protein (TBP), TBP-associated factors (TAFs), as well as co-factors, in a combinatorial way, which interact and form multi-protein complexes that can bind to either local and distal *cis-elements* (promoters and enhancers, respectively) of genes [37,38]. These complexes recruit and stabilize RNAPII at the promoter region, which ultimately leads to the preinitiation complex (PIC) formation upstream of the transcription start site (TSS). Additionally, these transcription factors may also facilitate the recruitment of chromatin modifiers to specific loci, consequently regulating gene expression in a spatio-temporal manner [38].

The phase of transcription **elongation**, which occurs after the initiation step, is characterized by its dynamic and intermittent nature and is subject to regulation by transcription elongation factors (TEFs) [39,40]. TEFs can be classified into different categories based on their functions [39,40]. These categories include regulators of RNA polymerase II (RNAPII) activity, effectors of epigenetic regulation (changes in gene activity without changes in gene sequence, such as histone chaperones and ATP-dependent chromatin-remodeling complexes), and enzymes responsible for writing or erasing covalent histone modifications within transcribed regions [39,40]. Following the elongation, the release of nascent RNA from the complex of RNAPII and DNA template occurs during the termination step in RNA synthesis [41,42]. The **termination** process is crucial in the regulation of gene expression as it can impact the stability and translation potential of RNA molecules [41,42]. The determination of the 3′ end of mature messenger RNA (mRNA) is attributed to the cleavage event, rather than the specific site where transcription comes to a halt [41,42]. Termination also plays a critical role in preventing un-regulated readthrough transcription of adjacent downstream genes. Numerous studies conducted over several decades have put forth various models, such as the allosteric/anti-terminator model (a model that states the termination of the RNA transcription process is caused by changes in structural conformations), the exoribonuclease 2 (XRN2)-mediated torpedo model (the termination of RNA transcription occurs due to the RNA degradation machinery catching up to the transcriptional machinery and subsequently displacing it), and a unified model that integrates both of these to elucidate the mechanism underlying polyadenylation signal (PAS)-dependent termination [41,42,43]. The phenomenon of RNAPII pausing downstream of the poly (A) site has been characterized in plants. This event promotes mRNA 3′-end processing and subsequent torpedo degradation for termination, which is mediated by the XRN2, FPA, a 3′-end processing factor, and BOUNDARY OF ROP DOMAIN1 (BDR1), a negative elongation factor [43,44].

An additional layer of eukaryotic transcriptional regulation is laid by the MEDIATOR (MED) complex. The mediator protein complex comprises approximately 34 subunits and acts as a molecular bridge between enhancer-bound gene-specific transcription factors and RNAPII [45,46,47]. The Mediator complex is a key component in the transcription process, as it remains centrally positioned and interacts directly with RNAPII, GTFs, and transcriptional activators/repressors that are activated by internal or external stimuli [45,46,47]. Moreover, the Mediator complex is involved in phosphorylating the carboxy-terminal domain of the largest subunit of RNA polymerase II, which is essential for initiating and maintaining gene-specific transcription. Specific Mediator family members fine-tune basic cellular processes such as cell growth and cell wall formation as well as numerous plant developmental aspects via crosstalk with phytohormone response pathways like auxin and abscisic acid (ABA) signaling involved in phase transition and flower development [45,46,47]. Furthermore, various animal systems have demonstrated the involvement of several components of the mediator complex in regulating tissue-specific energy metabolism in the liver, heart, and brain [48].

Transcriptional and post-transcriptional gene regulation are key aspects of sRNA-mediated RNA metabolism (Figure 2) [18]. Although small unstructured RNAs have been reported to potentially inhibit transcription in eukaryotes by interacting with RNA polymerase II (RNA Pol II) and preventing its binding to the DNA template, no related miRNA-mediated silencing activity of RNA polymerases has been reported yet [49]. Interestingly, MEDIATOR13 (MED13) has been reported to be negatively regulated by cardiac miRNA, miR-208a, and plays an important role in energy homeostasis [50]. Additionally, siRNAs can also inhibit RNA polymerase binding through TGS pathways by incorporating DNA methylation and chromatin marks, such as histone H3 methylation at lysine 9 (e.g., H3K9me1, H3K9me2, H3K9me3) in eukaryotes, thereby shifting the DNA template conformation from an open to a closed state. Numerous studies have demonstrated the role of lncRNAs as transcriptional regulatory factors. lncRNAs influence gene expression by modulating the accessibility of genes to RNA polymerase [51]. For example, lncRNA *Hidden treasure 1* (*HID1*) forms a nuclear protein-RNA complex and interacts with *Phytochrome-Interacting Factor 3* (*PIF3*) to suppress its transcription in Arabidopsis [52]. Similarly, the intronic lncRNA Cold Assisted Intronic noncoding RNA (*COLDAIR*) represses the transcription of the *Flowering Locus C* (*FLC*) gene by recruiting Polycomb Repressive Complex 2 (PRC2) to the *FLC* locus [53].

MiRNAs are known to target a wide range of transcription factors in both plants and animals, making miRNA-mediated transcriptional regulation more specific to target gene families, tissue types, as well as developmental stages. In addition to directly targeting, resulting in the silencing of the target TFs, miRNAs have also been shown to form complex interaction networks involving feedback and feedforward loops, as well as mixed interactions that involve both linear and loop interactions. For example, the miR165/166-*REVOLUTA* (*REV*) interaction forms a double negative feedback loop, where miR165/166 and *REV* mutually suppress each other. In contrast, the miR165/166-*ARABIDOPSIS RESPONSE REGULATOR1* (*ARR1*) interaction forms an incoherent feedforward loop, where both miR165/166 and *ARR1* co-regulate *PHABULOSA* (*PHB*) in a coordinated manner. These types of miRNA-mediated regulatory circuits have been extensively reviewed in [9]. miR-21, for example, is known to target phosphatase and tensin homolog (PTEN), an interaction that plays an important role in tumor suppression, cell growth, proliferation, and survival [54,55]. Other well-established and conserved miRNA-target modules involving TFs include miR-196 and miR-10 targeting Hox gene family members, which play a significant role in anterior-posterior body patterning, cellular identity, as well as cancer regulation [56,57,58,59]. In plants, numerous miRNA-target modules involving transcription factors have been identified, regulating key aspects of development, metabolism, and responses to both biotic and abiotic stresses, as detailed in Table 1. In addition to the extensive list of miRNAs targeting TFs, various siRNAs are also known to play similar roles. Notably, the miR390-*TAS3* tasiRNA and miR828-*TAS4* tasiRNA modules are highly conserved across the plant kingdom. The siRNAs generated from these modules specifically target ARF (such as *ARF2*, *ARF3*, and *ARF4*) and MYB transcription factor family members, respectively [60,61,62].

## 3. Intersections Between mRNA Decay and sRNA-Mediated Gene Silencing Pathways

Eukaryotic cells exhibit dynamic RNA turnover of mRNAs, which is important for growth and survival because it affects the abundance and composition of mRNA reservoirs. The principal factors that govern mRNA stability are the 5′ m7G cap and the 3′ poly(A) tail. Degradation of mRNA can occur in either the 5′→3′ or the 3′→5′ directions, and this process is carried out by two distinct groups of exonucleases, namely exoribonucleases (XRNs) and the exosome, which is a multi-subunit complex involved in RNA processing, turnover, and surveillance activities [43,103]. Based on the specific localization of the mRNA and the machinery responsible for its decay, mRNA decay can be classified into two distinct categories—nuclear decay and cytoplasmic decay.

**Cytoplasmic decay** is broadly characterized as deadenylation-independent, -dependent, and endonucleolytic cleavage-dependent decay. The deadenylation-dependent mRNA decay process is marked by a deadenylation mechanism that involves a three-subunit deadenylase complex comprising CCR4, CAF1, and NOT1 (also known as poly(A)-specific ribonuclease (PARN)) and a two-subunit Poly(A) Nuclease 2 (PAN2)–PAN3 deadenylase, which leads to a gradual reduction in the length of the 3′ poly(A) tails [104,105,106,107]. Subsequently, the mRNAs that have undergone deadenylation are directed towards either 3′→5′ decay via the exosome complex or the decapping process [108]. The exosome complex, consisting of six catalytically inactive 3′ → 5′ exoribonucleases in the core, associates with other subunits such as RRP6 (an RNase D 3′ → 5′ exoribonuclease localized in the nucleus and nucleolus) and/or RRP44/DIS3 (a processive RNase II 3′ → 5′ exoribonuclease localized in the cytoplasm and nucleus) and is catalytically activated [43]. After exosome-mediated 3′ → 5′ degradation in the cytoplasm, DcpS (DCS1 in yeast), a scavenger-decapping enzyme, hydrolyzes the remaining cap structure [109]. Decapping is facilitated by core decapping factors such as DECAPPING1 (DCP1), DCP2, and VARICOSE (VCS), which is then followed by 5′→3′ exonucleolytic digestion mediated by XRN1 (in animal systems)/XRN4 (in plant systems), which leaves a 5′ phosphate on the substrate [43,110], an important chemical feature exploited by researchers to interrogate the ‘degradome’ RNA profiles [111,112]. Co-translational mRNA decay can also serve as a regulatory mechanism for mRNA abundance, as demonstrated in *Arabidopsis thaliana*, a plant model system, as well as in human cell-lines [113,114]. This process involves the 5′→3′ XRN1/4 exonucleolytic degradation of mRNA sterically limited by stalled ribosomes, either due to the presence of a stop codon or by a miRNA binding site resulting in mRNA slicing or translational inhibition [113]. In addition, three primary pathways for mRNA quality control exist that degrade mRNAs in a translation-dependent manner. These pathways include nonsense-mediated decay (NMD), which targets mRNAs with premature termination codons (PTCs); non-stop decay (NSD), which targets mRNAs lacking stop codons; and no-go decay (NGD), which targets mRNAs with stalled ribosomes in the coding region [115]. The fundamental machinery of NMD is comprised of three Up-frame shift (UPF) proteins, namely UPF1, UPF2, and UPF3. RNA splicing involves the participation of UPF3 leading to the formation of the exon-exon junction complex (EJC) [115]. This complex is attached to spliced mRNAs that are transported to the cytoplasm. In transcripts containing a PTC, EJCs may remain bound to mRNAs during translation, leading to Nonsense-Mediated Decay (NMD) initiation in an SMG/UPFs-dependent manner [115,116]. In addition to NMD, the two other translation-dependent pathways of mRNA surveillance, namely NGD and NSD, have been found to be associated with the Superkiller (SKI) complex (SKI2, SKI3, SLI7, and SKI8) [115]. The SKI complex facilitates the exosome-mediated degradation of cytoplasmic mRNAs from their 3′ ends [115]. In addition, ABSCISIC ACID HYPERSENSITIVE 1 (ABH1)/CAP BINDING PROTEIN 80/CBP80 interacts with LOW-LEVEL BETA-AMYLASE 1 (LBA1), which is required for NMD that ensures transcripts with premature termination codons are degraded [117]. **Nuclear mRNA decay mechanisms** are 5′ → 3′ and 3′ → 5′, similar to those found in the cytoplasm, and are crucial for overall RNA turnover. The TRAMP complex destabilizes nuclear RNAs, including rRNAs, through polyadenylation, resulting in accelerated exosome-mediated 3′ → 5′ decay, in contrast to cytoplasmic mRNAs that are stabilized by polyadenylation. The nuclear exosome degrades aberrant transcripts that have not been spliced or incorrectly polyadenylated [43]. In yeast, XRN2 (RAT1) degrades nuclear-restricted mRNAs after being decapped by LSM2–8 proteins [118]. Evidence from single-molecule nascent RNA sequencing shows the cleavage occurring at the poly(A) site serves as a point of entry for the 5′→3′ exonuclease, AtXRN2/3, in *Arabidopsis thaliana*, which degrades the 3′ cleavage product [44].

**Figure 3:**
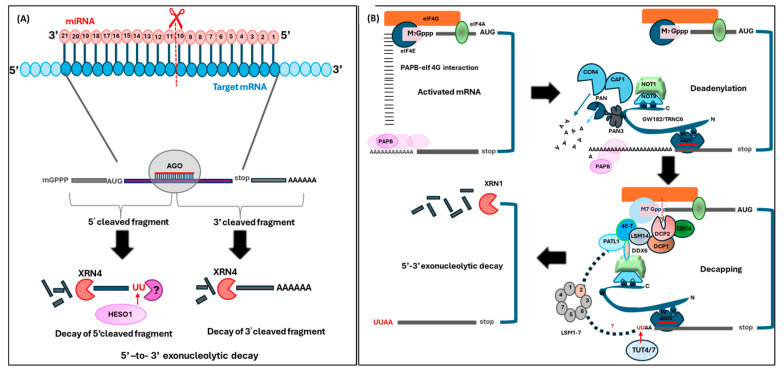
miRNA-mediated mRNA decay pathway in plants and animals. (**A**) miRNA binds to the complementary site in the Open Reading frame and induces endonucleolytic cleavage at the splice site (between the nucleotides 10 and 11). The 5′ fragment is uridylated by HUA Enhancer 1 Suppressor 1 (HESO 1) and is degraded by XRN4 in a 5′ to 3′ direction. Similarly, the 3′ cleaved fragments are degraded by XRN4 without uridylation. (**B**) miRNA, after attaching with the activated mRNA, recruits CCR4-NOT and PAN2-PAN3 deadenylase complexes to target mRNAs via the GW182 protein. These deadenylated mRNAs are then oligouridylated by TUT4/7, thus starting the general mRNA decay in mammals. Apart from deadenylation, GW182 can also promote dissociation of PAPB (poly(A) binding protein). DDX6 (the de-capping activators) are then recruited onto the CCR4-NOT complex. This helps the DCP2 enzyme in removing the 5′ 7-methylated guanine cap. Finally, XRN1 acts on the uncapped uridylated mRNA strand by performing 5′-3′ exonucleolytic decay. (Modified from [127]).

Apart from the above-mentioned mRNA decay pathways, sRNA-mediated PTGS and TGS pathways also directly influence the mRNA decay pathways, as miRNA-sliced mRNA transcripts are known substrates of XRN1/4-mediated degradation (Figure 3) [119,120]. Aberrant transcripts, such as those derived from over-expressed transgenes, viral RNA, and a subset of endogenous genes like transposable elements or those that have escaped the above-described degradation pathways, can then be converted to dsRNAs that are PTGS triggers by the biosynthetic action of RNA-dependent RNA polymerases (RDRs) and/or coiled-coil domain-zinc finger SUPPRESSOR OF GENE SILENCING 3 (SGS3) and novel plant-specific SILENCING DEFECTIVE 5 (SDE5) (Figure 1) [121]. Numerous studies have demonstrated that inhibition of undesired endogenous PTGS relies on decapping activities and the convergence of bidirectional cytoplasmic mRNA decay pathways [115,122,123]. This is evidenced by the fact that the pleiotropic phenotypes of several mRNA decay pathway mutants, including *xrn4*, *ski2*, and *dcp1*/*2,* are rescued by PTGS pathway mutants such as *rdr6*, *dcl2*, and *dcl4* [110,124,125]. In addition, mutations in *abh1* result in an increase in the quantity of XRN4-affected sRNAs, presumably because destabilization of the 5′ end of mRNA, as well as accumulation of unspliced mRNAs, leads to increased exosome/XRN4-mediated aberrant mRNA degradation, resulting in an increase in the quantity of sRNAs [1,126]. On the other hand, animal miRNAs can enhance mRNA decay not only by recruiting deadenylases to the target mRNAs via the GW182 protein (known as TNRC6A-C in mammals and GW182 or Gawky in Drosophila) but also by making the poly(A) tail more accessible to these enzymes. The GW182 protein interacts with two deadenylase complexes, CCR4–NOT and PAN2–PAN3, through tryptophan (W) motifs in its C-terminal silencing domain. Recent research suggests that the CCR4-NOT complex is more pivotal in miRNA-mediated deadenylation and mRNA decay than the PAN2-PAN3 complex [11,127]. sRNAs not only initiate the degradation of target RNAs but also contribute to the biogenesis of additional sRNAs during the decay process, generally referred to as transitivity. This cyclical mechanism establishes a negative feedback loop, where sRNA-triggered RNA degradation can lead to the production of new sRNAs, thereby maintaining the homeostatic RNA pool within a cell at any given time.

## 4. Crosstalk Between Nascent RNA Processing, RNA Modification, and sRNA Pathways

Nascent RNAs synthesized by RNAPII undergo multiple processing steps before maturation. These include splicing of introns—a process mediated by the spliceosome complex, the selection of 3′ end sites, and adding a poly(A) tail of variable lengths. Additionally, the Exon Junction Complex (EJC) and Transcription/Export (TREX) complex are involved in transporting these RNAs, ensuring their proper export from the nucleus to the cytoplasm [4]. mRNA processing events like 3′ poly(A) tail, 5′ m7G cap, and alternative splicing (AS) are known to play a significant role in increasing the stability of an mRNA as well as the export kinetics of the mature mRNA from nucleus to cytoplasm before translation. Interactions of RNAs with myriad effectors, including those involved in RNA processing, can occur sequentially and/or simultaneously during transcription (co-transcriptionally), thereby exerting mutual influence [128]. Nuclear sRNAs can target nascent RNA molecules from RNAPs, resulting in co-transcriptional gene silencing (CTGS) by adding epigenetic marks at loci undergoing active transcription [18]. Thus far, two theoretical frameworks have been proposed to elucidate the mechanisms of nascent RNA processing coupling in eukaryotic systems. The first model, referred to as the recruitment model, posits that transcription plays a pivotal role in linking various RNA processing events, primarily mediated by RNAPII [129]. The recruitment of diverse processing factors to a nascent RNA, such as capping factors, splicing factors, and polyadenylation factors, is facilitated by RNAPII acting as a platform [129]. The CTD of RNAPII (made up of Y_1_S_2_P_3_T_4_S_5_P_6_S_7_ tandem repeats), upon phosphorylation at serine 5 (Ser5P), exhibits a specific association with the spliceosome complex during co-transcriptional splicing. In addition to RNAPII, chromatin state is also a substrate variable for binding by processing factors and is often dictated by siRNAs-guided deposition of methylation marks. For instance, RBPs can be recruited by histone H3 tri-methylated at lysine 36 (H3K36me3), while the U2 small nuclear ribonucleoprotein (snRNP) can be recruited indirectly to promote splicing by H3K4me3 [129,130]. The second model, referred to as the kinetic model or kinetic competition model, posits that the output of transcript isoforms and the relative content of various isoforms can be influenced by the relative rates of transcription elongation and splicing or poly(A) site cleavage [13]. The elongation rate of RNAPII is comparatively low, which facilitates the functioning of RNA processing factors. This is because the slow rate allows for a longer duration for the assembly of the spliceosome, binding of processing factors, and identification and cleavage at specific poly(A) sites [13]. As the transcription elongation rate may affect the recruitment of various processing factors, which in turn may affect the transcription elongation rate via differential recruitment of elongation rate-controlling factors, the possibility of both models being inter-dependent is yet to be explored [13].

**Splicing or alternative splicing** plays a significant role in increasing the functional diversity of proteins encoded by a single mRNA but translated differently at a specific time and space [131]. Alternative splicing (AS) not only expands the coding potential of intron-retaining genes but also plays a key role in gene regulation through mechanisms such as nonsense-mediated decay (NMD) and miRNA-mediated regulation [12]. Loss of splicing factors may lead to loss of sRNA biogenesis, leading to the transcriptional activation of the sRNA targets, or it can also lead to increased production of sRNAs, leading to increased repression of the respective targets throughout the genome. A forward genetic screen in *Schizosaccharomyces pombe* identified mutations in essential splicing factors such as Cwf10 [132], a U5 small nuclear ribonucleoprotein homolog, and Prp39 [133], a human PRPF39 homolog, both of which are critical for stable pre-mRNA and spliceosome component interactions. These mutations led to the activation of a reporter gene in the normally siRNA-mediated transcriptionally repressed repetitive outer sequences (otr) region [132]. Effects on the sRNA pathways observed in a splicing factor mutant lacking *cwf14* could be partially rescued by the introduction of properly spliced cDNAs encoding key sRNA machinery components, such as AGO1, suggesting that the observed defects may be attributed to faulty splicing of sRNA-related transcripts [134]. In addition, Cid12, a key component of the RNA-directed RNA polymerase complex involved in RNA processing and siRNA biogenesis, has been shown to physically associate with a large proportion of spliceosome complex components. This association evidence provides a direct link between splicing factors and the canonical sRNA pathway machinery, suggesting that defects in splicing are likely to impact sRNA-mediated silencing pathways [132]. Many plant MIRNA genes contain introns, and the splicing of these introns, which are generally located downstream of the first exon in pri-miRNAs, is crucial for regulating pri-miRNA processing and miRNA biogenesis. The assembly of the spliceosome at the 5′ splice site influences the length and structure of MIR primary transcripts, thereby affecting mature miRNA levels. In addition, this splicing process modulates miRNA abundance in response to various stimuli, including biotic factors (such as pathogen-induced accumulation of mature miR163 [135]), abiotic stressors (like heat-induced accumulation of mature miR400 [136]), and hormonal signals (such as ABA-mediated regulation of mature miR846 and miR842 [137]). Moreover, mutations in genes encoding proteins involved in RNA processing, like 3′ end formation (ENHANCED SILENCING PHENOTYPE1/4/5 (ESP1/4/5); components of cleavage polyadenylation specificity factor (CPSF)), splicing (ESP3; homologue of Pre-mRNA-processing protein 2 (PRP2)), were identified in a screen aimed at characterizing mutants with enhanced silencing phenotypes (*esp*) in *A. thaliana*. While the intron splicing efficiency is crucial for the generation of aberrant RNAs that serve as templates for sRNA biogenesis, the presence of introns in foreign sequence transgenes can also reduce the likelihood of transgene silencing by RNAi mechanisms [138].

Numerous **mRNA chemical modifications** have been identified in mammalian and plant mRNAs. These include an N7-methylguanosine (m^7^G) 5′ cap on a protective triphosphate 5′-end structure, N6-methyladenosine (m^6^A), 3,4-N6-2′-O-dimethyladenosine (m^6^Am), N1-methyladenosine (m^1^A), 6, 7, 5-methylcytosine (m^5^C), 5-hydroxymethylcytosine (hm^5^C), 2′-O-methylated nucleosides (Nm), inosine (I), pseudouridine (Ψ), and uridylation modifications [139,140,141,142,143,144,145,146,147,148]. All the above-mentioned modifications during pre-mRNA processing can affect mRNA metabolism and function in a cellular context. In addition to mRNAs, sRNAs undergo 2′-O-methylation (Nm) by the sRNA 2′-O-methyltransferase HEN1, which is conserved across kingdoms. This methylation protects sRNAs from 3′-uridylation and 3′-truncation, both of which can lead to sRNA degradation. The specificity of HEN1 may also contribute to cell-type-specific sRNA profiles and influence the determination of RNAi targets; however, the underlying credibility of this process has yet to be uncovered [149,150]. Chemical modifications, such as m6A in 5′ and 3′ UTRs, have the potential to modify the expression fate of mRNAs by inducing changes in their physical properties and affecting their fate by regulating splicing, export, decay stabilization, and translation. Alternatively, m6A writers, like METHYLTRANSFERASE-LIKE 3 (METTL3) and METTL14, and additional co-factors, like WTAP, KIAA1429, ZC3H13, RBM15, and METTL16, mediated modifications that result in the recruitment of regulatory proteins referred to as m6A readers, which possess a distinct YTH mixed alpha/beta-fold domain conserved across all eukaryotes that directly binds to m6A [151]. m6A reader proteins, namely EVOLUTIONARILY CONSERVED C-TERMINUS 2 (ECT2), ECT3, and ECT4, homologs of the YTHDF protein family in humans, exhibit cytoplasmic localization and regulate the expression of m6A-modified genes in a spatiotemporal manner by recruiting additional proteins, like poly(A) binding (PAB) proteins [152]. While m6A alterations can influence AS events, and AS events can influence the efficacy of m6A readers-mediated gene regulation, this reciprocal interaction between m6A readers and AS events creates a feedback loop that allows them to mutually control one another [153,154]. During miRNA biogenesis, pri-miRNAs undergo a series of cleavage steps to convert into pre-miRNAs and eventually mature miRNAs. This process is tightly regulated by various factors, including RNA methylation, and it has been shown that pri-miRNAs are often enriched with the m6A motif (GGAC), which allows them to undergo m6A modification mediated by METTL3/14. Heterogeneous nuclear ribonucleoprotein A2B1 (HNRNPA2B1), by recognizing m6A marks, facilitates miRNA processing through the DiGeorge syndrome critical region 8 (DGCR8) complex [155]. Recent studies have revealed that m6A methylation is crucial for maintaining proper levels of mature miRNAs and their precursors in *Arabidopsis thaliana*. This regulation occurs through the biogenesis pathway, where m6A interacts with RNA Pol II, and the export pathway, involving interaction with TOUGH. Furthermore, m6A marks play a role in recruiting the microprocessor complex to pri-miRNAs [156]. Given that both alternative splicing (AS) and m6A events can influence sRNA production and activity, there is potential for an additive effect, though this hypothesis remains untested in plants.

## 5. RNA Editing, Retrograde Signaling, and Potential Links to sRNA Pathways

RNA editing is a vital supplement to the central dogma that occurs co-transcriptionally as well as post-transcriptionally, which involves the alteration of primary transcripts through introducing nucleotide indels (insertions or deletions) or substitutions, yielding genetic information in RNA products different than the DNA template for transcription [157]. Various forms of RNA editing such as the conversion of cytidine (C)-to-uridine (U), U-to-C, and adenosine (A)-to-inosine (I), insertion or deletion of U, as well as insertion of guanosine (G), have been extensively characterized in a range of organisms such as protozoa, viruses, humans, mice, zebrafish, and plants [158,159,160]. RNA editing, rectifies defective organellar transcripts by restoring conserved codons or creating start or stop codons and has been well-documented in various members of the plant kingdom, including Arabidopsis, rice, wheat, tobacco, maize, and soybean [161,162,163,164,165]. In plants, mitochondrial and plastidial transcripts are generally subjected to RNA editing, and the predominant mode of RNA editing in plants is the conversion of C-to-U. The phenomenon of RNA editing was first reported in wheat mitochondria [166]. Two years later, Hoch and colleagues provided additional evidence indicating that the conversion of a typical ACG codon to an AUG initiation codon in the mRNA of the *rpl2* gene in the maize plastids was attributed to C-to-U editing [167]. Members of the PLS subfamily of pentatricopeptide repeat (PPR) proteins are the key genetic factors responsible for site-specific RNA editing in chloroplasts and mitochondria [168,169]. In addition to PPR proteins involved in site-recognition, further crucial constituents of RNA editing complex(es) include MULTIPLE ORGANELLAR RNA EDITING FACTORS (MORFs), ORGANELLE RNA RECOGNITION MOTIF (ORRM) proteins, ORGANELLE ZINC-FINGER (OZ) proteins, and PROTOPORPHYRINOGEN OXIDASE 1/PPO1 [170,171,172,173,174]. Recently, via direct interaction of GENOMES UNCOUPLED 1 (GUN1) and MORF2, a link has been established between RNA editing and chloroplast retrograde signaling pathways in plants, highlighting an emerging area of overlap between these processes [175].

Chloroplasts and mitochondria are the semi-autonomous organelles that synthesize food and energy, respectively, within plant cells. These organelles are essential for plant cell survival and play significant roles in sensing environmental stimuli and adaptive stress responses. As semi-autonomous organelles, chloroplasts and mitochondria have distinct genomes that encode approximately 80–100 and 20–40 protein-encoding genes, respectively [176,177,178]. Sigma factors of the plastid-encoded RNA Polymerases are encoded by the nuclear genome and must be coordinately expressed to the respective organelles through a process known as anterograde signaling in order for chloroplast and mitochondrial machinery to develop and function in a regulated and effective manner [179]. In addition, as the ultimate ‘end game’ subjects of plant stress response pathways, chloroplasts and mitochondria can regulate the expression of nuclear genes under stress conditions through a process known as retrograde signaling [32,180,181,182,183,184]. Several retrograde signals of chloroplasts have been identified, specifically the redox state of plastoquinone (PQ), stress-activated RNA editing status of chloroplast genes, 3′-phosphoadenosine 5′-phosphate (PAP), carotenoid derivatives, reactive oxygen species (ROS), and precursors of isoprenoids such as methylerythritol cyclodiphosphate (MEcPP) [180,182,185,186,187]. Stress-activated GUN1-MORF2-PPR complex changes the **RNA editing** status of chloroplast genes, acting as a retrograde signal for activating or suppressing nuclear gene expression [175]. By comparison to chloroplast retrograde signals, mitochondrial retrograde signals are relatively unknown. It has recently been demonstrated that mitochondrial retrograde signaling facilitates proper mitochondrial function via *NAC DOMAIN CONTAINING PROTEIN 17* (*ANAC017*), cellular stress responses such as hypoxia mediated by *UNCOUPLING PROTEIN 1* (*UCP1*), and responses to ethylene during germination mediated by *ALTERNATIVE OXIDASE 1A* (*AOX1a*), a mitochondrial retrograde sign aling marker) and ANAC013 [184,188]. On the other hand, ROS production can serve as a signal for both mitochondrial and chloroplast retrograde signaling pathways.

Recent research has uncovered that small mitochondrial highly expressed RNAs (smithRNAs), first identified in *Ruditapes philippinarum* and transcribed from the mitochondrial genome, have the ability to regulate gene targets encoded in the nuclear genome [189]. In addition to presenting evidence showing interaction between smithRNAs and AGO2, a key RNAi component in animals, Pozzi et. al., 2022, also highlight cross-species conservation of smithRNAs encoded within the mt-tRNA Met gene across Chordata [190]. This finding fuels the possibility of direct involvement of sRNAs in retrograde signaling pathways in both animals and plants. Recently, accumulation of certain plant miRNAs, like miR157a, miR169g-3p, miR395b/c, miR398, miR850, miR863, and miR5026, as well as natural cis-antisense siRNAs (cis-nat-siRNAs), was found to be misregulated in *gun* (*gun1* and *gun5*) mutants [191]. Moreover, direct and indirect effects of chloroplast retrograde signaling on RNA metabolism have also been established. For example, PQ-mediated chloroplast retrograde signals regulate RNA metabolism by influencing AS of nuclear genes. This regulation occurs through the modulation of RNAPII elongation rates in response to light-induced changes in the redox state of photosynthetic electron transport components [32]. Moreover, PAP, which is regulated by tocopherol metabolism, inhibits the activity of *XRN2,* which directly affects RNA metabolism at post-transcriptional levels by enhancing sRNA accumulation [192]. Furthermore, retrograde signals, like ROS, can also influence sRNA accumulation, including miR398 [193], miR400 [194], miR408 [195,196], miR528 [197], which can lead to post-transcriptional silencing of the respective targets. Collectively, these findings establish a loop where RNA metabolism plays a crucial role in the transduction of retrograde signals, and retrograde signaling, in turn, modulates RNA metabolism, particularly through mechanisms like AS.

## 6. The Role of RNA Metabolism and sRNA-Mediated Gene Regulation in Plant Growth, Development, and Stress Responses

RNA metabolism is a multistep integrated process in which the flow of genetic information can be controlled at each stage. Misregulation at any step, whether RNA synthesis/translation or RNA decay, can disrupt the linear flow of genetic information from DNA to proteins, resulting in mild to severe pleiotropic developmental defects and embryonic lethality. Decades of research have shown that RNA metabolism regulation is an important factor in cellular and developmental processes such as stem cell maintenance, vascular development, vegetative and reproductive organ development, root development, flowering time, circadian clock, light signaling, and disease resistance and stress responses (reviewed in [1,3,198,199,200,201])

The regulation of flowering time and the transition from vegetative to reproductive phases via RNA metabolism regulation are well described. The transition from the vegetative to reproductive phase is initiated by the suppression of *FLOWERING LOCUS C*/*FLC/AGAMOUS-LIKE 25* expression through an AS mechanism mediated by glycine-rich RNA-binding proteins (GRPs) and RZ-1A-C, and by alternative polyadenylation of *FLC* pre-mRNA by its antisense transcript *COOLAIR,* facilitated by AS of RBD-encoding *FLOWERING CONTROL LOCUS A/FCA* mediated by FPA, FY, hnRNP A1-like protein 1 (HLP1), and cleavage and polyadenylation specificity factor CPSF100. Furthermore, the promotion of flowering is facilitated by the stabilization of Phosphatidylethanolamine-binding protein transcript *FLOWERING LOCUS T/FT* (aka ‘Florigen’) and TFs *SQUAMOSA PROMOTER BINDING PROTEIN-LIKE 3*/*7* (*SPL3*/*7*) mRNA through m6A demethylation [3,143,202,203,204,205]. Moreover, ORRM4-mediated mitochondrial RNA editing, ALKBH10B (a m6A eraser), XRN4, and PAPS1 (a nuclear canonical poly[A] polymerase) also regulate flowering time and flower development [43,143,206,207]. Regulation of flowering time is influenced by transcription elongation factors such as P-TEFb, HUB1, UBC1, SUP32/UBP26, ATXR3/SDG2, ASHH2, the Polymerase-associated factor 1 complex (PAF1C), JMJ14, LSD1, and SWR1C. These factors interact directly with RNAPII, thereby modulating transcription efficiency or gene-specific epigenetic signatures, ultimately impacting gene expression [208].

Broader involvement of RNA metabolism effectors in the growth and development of monocots and dicots is revealed by molecular genetic analysis of *U11*/*U12*-*31K*, which is one of seven RNA chaperone gene products present in the minor U12 intron spliceosomal complex [209]. *JULGI*-mediated RNA folding, which results in the formation of RNA G-quadruplexes in the 5′ UTRs of *SUPPRESSOR OF MAX2-LIKE1*-*4*/*5* (*SMXL4*/*5*), regulates the translational status of *SMXL4*/*5* mRNAs and has been identified as a key positive regulator of phloem development [210]. The modulation of root development through the differential alternative splicing of auxin-responsive genes is facilitated by nuclear speckle RBPs (NSR) 1 and 2, which are plant-specific proteins that possess a C-terminal RNA recognition motif domain. These proteins form a regulatory module that competes for interaction with alternative splicing targets versus structured RNAs such as *EARLY NODULIN 40* (ENOD40) and *long non-coding lnc351/Alternative Splicing Competitor/ASCO* [211,212]. The regulation of seed dormancy, vegetative and reproductive development, photomorphogenesis, developmental patterning, and embryo basal-apical polarity is significantly influenced by transcription elongation factors such as TFIIS, SPT4/5/6, P-TEFb, FACT, HUB1, REF6/JMJ12, and SWR1C [208]. The regulation of transcription through the Mediator complex has been demonstrated to play a significant role in modulating fundamental cellular processes such as cell growth and cell wall formation. Specifically, MED25, MED8, MED16, MED33A, and MED33B have been identified as key components in this process [47]. Additionally, the Mediator complex has been implicated in regulating developmental processes, including the network of MED8, MED12, MED13, MED16, MED18, and MED25 [47]. Notably, the Mediator complex has also been shown to interact with phytohormonal signaling pathways, such as those involving auxin and abscisic acid, which are known to be critical in phase transition and flower development [47]. In addition, RNA editing in organelles has the potential to result in developmental defects in plants (reviewed in [157]).

RNA metabolism also plays a key role in stress responses. Stress-responsive RBP phosphoprotein INVOLVED IN RRNA PROCESSING 2/AtLa1 mediates 5′ cap-independent translation initiation of the meristem maintenance homeobox TF *WUSCHEL* (*WUS*) mRNA by interacting with an internal ribosomal entry site. DEAD-box RNA helicases (RHs) are significant contributors to various stress responses, including cold, salinity, heat, and osmotic stresses [213,214]. This is achieved through their ability to catalyze the unwinding of secondary structures of RNAs, thereby influencing RNA metabolism within a cell. Transcription elongation factors, namely SWR1C, REF6/JMJ12, ASHH2/SDG8, and HUB1, are important for diverse biotic and abiotic stress responses [208].

miRNA-mediated PTGS fine-tunes the expression of key effectors that regulate different aspects of plant development and responses to abiotic and biotic stressors. Table 1 highlights a subset of these conserved interactions between miRNAs and TFs. To complement the above-mentioned pathways of RNA metabolism, deeply conserved antagonistic interactions between miR156:*SPLs* and miR172:*AP2* domain transcription factor PTGS modules also regulate age-dependent flowering time in all flowering plants [215]. Additionally, miRNA-TF modules, like miR159:*MYB*s, miR172:*AP2*, miR164:*NAC*s, and miR167:*ARF*s, also regulate various aspects of flower development, including gynoecium and androecium development, meristem boundary identity, and floral organ development (see Table 1). Beyond flowering time and flower development regulation, certain miRNA-TF modules are also essential for leaf, root, shoot, embryo, and fruit development, as well as stress response (see Table 1). Other than targeting TFs, plant miRNAs also target other protein-coding genes, and these interactions are of equal importance. For example, miR162, miR168, and miR403 are known to target *DCL1*, *AGO1*, and *AGO2*, respectively, genes that are key in regulating miRNA biogenesis as well as sRNA-PTGS activities and thus have pleiotropic effects on plant development (reviewed in [29]). Certain miRNA-target interactions, like miR395:ATP sulfurylases, miR399:*PHO*2, miR827:*SPX,* miR398:*CSD*s, and miR408:*PLC*/*ULC*/*Cupredoxin*, are involved in regulating metal ion homeostasis, including sulfur, phosphate, and copper metabolism, respectively (reviewed in [29]). Furthermore, miRNA-target modules, like miR161:*PPR*s, miR163:*SABATH,* and miR482/2118:*NBS-LRRs*, participate in abiotic and biotic stress responses (reviewed in [29]). Notably, the miR482/2118:*NBS-LRRs* module is distinct from the others, as members of the miR482/2118 family initiate phasiRNA production from *NBS-LRRs*, amplifying the silencing effect on their targets. While miRNAs play a crucial role in regulating overall plant development, siRNAs stand out for their functional significance in plant immunity. siRNAs are integral to the plant’s defense mechanisms, particularly in targeting a wide range of viral, bacterial, and fungal pathogens. By degrading foreign genetic material and silencing pathogen genes required for infection, siRNAs strengthen the plant’s immune response. Moreover, RDR1/6-dependent siRNAs are crucial for regulating intracellular immune receptors, playing a vital role in modulating broad-spectrum resistance [216]. siRNAs also strengthen plant immunity by ensuring genome stability and controlling the expression of immune-related genes via RdDM and TGS pathways [15,17]. Additionally, siRNAs generated from miRNA-targeted *TAS* loci and phasiRNAs play significant roles in both plant development and stress responses [60,200,217,218,219]. For example, tasiRNAs are generated from the interaction between miR173 and *TAS1* targets *HEAT-INDUCED TAS1 TARGET1* (*HTT1*) and *HTT2*, which regulates thermotolerance in *A. thaliana* [220]. miR1509:*TAS-LIKE* (*TASL*), miR173:*TAS1/2*, and miR7122:*TASL1/2*-derived tasiRNAs are also known to target members of the PPR family, which has broader roles in RNA metabolism, organelle development, and stress responses [221,222,223]. Furthermore, miR390:*TAS3*-derived tasiRNAs and miR393:*TIR1*/*AFB2*-derived phasiRNAs are crucial for leaf development, while miR828:*TAS4*-derived tasiRNAs significantly regulate secondary metabolism in plants [61,223,224].

## 7. Conclusions and Future Directions

In living organisms, DNA serves as the genetic blueprint, while RNA transfers the genetic information from DNA to proteins. Although the hypothesis that RNA predates DNA and proteins in evolution is widely accepted, its significance is often underappreciated in comparison to DNA and proteins, particularly from an industrial perspective. This is largely due to RNA’s inherent instability and susceptibility to degradation during processing and handling, leading to greater technical variability and challenges in its application. However, the recent COVID pandemic has highlighted RNA’s importance, as many people have become aware of the RNA, RNA-based genomes, and RNA-based vaccines.

Cellular RNA biology is not just limited to the transcription but also includes an intricate network of pathways with which RNA molecules are processed, modified, edited, mobilized, stored, degraded, and selectively utilized by the translational machinery for protein formation. Collectively, these pathways contribute to what is known as “RNA metabolism”. Although significant progress has been made in understanding the mechanisms contributing to RNA flux, our knowledge of RNA metabolism is still far from complete. For instance, while it is known that light and chloroplast retrograde signaling regulate splicing in leaves, the mechanism by which light influences splicing in roots remains unclear. Additionally, certain mRNAs show selective differential abundances, whereas others maintain steady levels, raising further questions about the regulatory mechanisms governing RNA stability and abundance. Furthermore, gene regulatory networks involved in RNA metabolism are highly interconnected. Any misregulation at a specific point within this intricate web can have far-reaching consequences, resulting in pleiotropic effects, potentially leading to cell death. Due to this pleiotropic nature of RNA metabolism effectors, it is difficult to perform gene function studies using reverse genetics practices. The newest generation of single-cell and spatial platforms, combined with reverse genetics tools, offers a significant advantage for investigating the functions of RNA metabolism effectors in specific cell types, cell states, and developmental stages [225,226,227].

Over the past two decades, progress in bioinformatics and sequencing technologies has deepened our understanding of gene regulation mechanisms across organisms while highlighting the conserved traits of the RNA world. These advancements have also led to the discovery of a new generation of RNA molecules, such as lncRNAs, circRNAs, and smithRNAs [19,23,189]. However, the exact nature and functional significance of many RNA molecules remain poorly understood. This gap in knowledge could also be bridged by leveraging recent sequencing technologies capable of measuring the qualitative and quantitative aspects of the transcriptome at both single-cell and spatial levels [225,226,228,229,230]. In addition, applying advanced AI and machine learning (AI/ML) models to high-depth, high-dimensional datasets with precise cellular and spatial contexts can significantly enhance biological discoveries. These models help reduce noise and technical variation, enabling more accurate measurement of the characteristics of the cellular transcriptome [231,232,233].

sRNAs are crucial regulators of RNA metabolism, playing a multifaceted role in shaping and fine-tuning the transcriptomic landscape. The majority of sRNA investigations thus far have centered on examining the spatiotemporal expression patterns of sRNAs and their targets in various tissues and environmental conditions, with the aim of elucidating the intricacies of sRNA-mediated developmental processes and environmental adaptations. The functional limits of the sRNAome are currently a topic of debate, due in large part to spurious claims for non-canonical sRNA activities based solely on homology predictions, since canonical sRNA:target interactions are stereotyped on high sequence complementarity in the ‘seed’ region, spanning nucleotides 2–13 in plants and 2–7 in vertebrates [234,235]. Beyond complementarity, free energy of the sRNA:target duplex and target site accessibility are also considered as important parameters for identifying sRNA:target interactions and minimizing false positives. However, studies have demonstrated that these parameters alone are insufficient to reliably predict true sRNA:target interactions in a genome [236]. Recently, Tjaden, B. (2023) demonstrated that high sequence complementarity in the seed region alone cannot distinguish the true sRNA:target interactions from non-interactions (false positives) [237]. By evaluating 111 features (a high-dimensional data input), including sequence complementarity in seed regions of variable lengths, Tjaden, B. (2023) identified additional features like presence as well as the number of sRNA:target homologs (cross-species conservation), sRNA:target duplex energy, length of the target coding region, and characteristics of the upstream sequence from the target start codon, which improved the ability of their machine learning model (Gradient Boosting) to differentiate the true sRNA:target interactions from the non-interactions. This study highlights the potential of utilizing and leveraging advanced AI/ML computational approaches and incorporating a broader range of features beyond just seed region sequence complementarity to achieve high-confidence identification of sRNA interactions in future research.

## Figures and Tables

**Table 1:** List of miRNAs and respective transcription factor targets in plants.

miRNA	Target Transcription Factor Family	Target Tissue/Cells	Regulatory and Developmental Function	References
miR156/157	*SPL* gene family	seed, leaf, root, stem, trichome, pistil, and nodule	Vegetative to reproductive phase transition, leaf and root development, abiotic stress response, secondary metabolism	[63,64,65]
miR159	*GAMYB* or *GAMYB-like* gene	seed, leaf, root, stamen, and pollen	Seed, leaf, and male reproductive development, drought response	[66,67]
miR160	*ARF10/16/17*	seed, leaf, stem, root, stamen, stamen, and pollen	Development of embryos, leaves, and roots, hypocotyl elongation	[68,69,70]
miR164	*NAC* gene family	seed, leaf, lateral root, shoot apical meristem (SAM), flower, and fruit	Boundary formation, leaf, lateral root, fruit and flower development, auxiliary meristem formation	[71,72,73]
miR165/166	*HD-ZIPIII* gene family	seed, root, SAM, vascular bundles, and nodule	Maintenance of meristematic cells, adaxial identity of leaves, promotion of the lateral root growth, and procambium development	[74,75,76]
miR167	*ARF6/8*	root, stamen, and pollen	Embryo development, root, stem, leaf, and flower formation, flowering time, abiotic stress response, pathogen defense	[77,78,79]
miR169	*CBF* and *NF-YA* family	leaf, root, flower, and nodule	Flower and root development, abiotic stress response	[80,81,82]
miR170/171	*SCARECROW*-like transcription factor genes	embryo, SAM, stem, leaf, and root	leaf, root, and flower development, meristem formation and maintenance, chlorophyll biosynthesis, phase transition	[83,84]
miR172	*AP2*, *TOE1/2/3*, *SMZ*, *SNZ*	leaf, SAM, and flower	Flower meristem identity and organ development, vegetative to reproductive phase transition	[85,86,87]
miR319	*TCP2/3/4/10/24*, *MYB33*/*65*/*81*/*97*/*104*/*120*	SAM, leaf	leaf development and senescence, abiotic stress response, plant architecture, and grain yield	[88,89,90,91]
miR393	*AFB*	embryo, root, shoot, leaf	Participates in embryo, root, shoot, and leaf development; biotic and abiotic stress response	[92]
miR396	*GRFs*	embryo, SAM, leaf, and lateral root	somatic embryogenesis, leaf growth, flower development, grain size, panicle branching, biotic and abiotic stress response	[93,94,95,96,97]
miR828 and miR858	*MYB*s	leaf, stem, flower, and fruit	Fiber development, lignin biosynthesis, trichome development, anthocyanin, and flavanol accumulation	[61,98,99,100,101,102]

## Data Availability

Not applicable.
